# Insights into metastatic roadmap of head and neck cancer squamous cell carcinoma based on clinical, histopathological and molecular profiles

**DOI:** 10.1007/s11033-024-09476-8

**Published:** 2024-04-29

**Authors:** Nicholas S. Mastronikolis, Alexander Delides, Efthymios Kyrodimos, Zoi Piperigkou, Despoina Spyropoulou, Evangelos Giotakis, Evangelos Tsiambas, Nikos K. Karamanos

**Affiliations:** 1 Department of Otorhinolaryngology – Head and Neck Surgery, School of Medicine, University of Patras, Patras, 26504 Greece; 2 2nd Otolaryngology Department, School of Medicine, National & Kapodistrian University of Athens, ‘Attikon’ University Hospital, Rimini 1, Athens, 12462 Greece; 3 1st Otolaryngology Department, School of Medicine, National & Kapodistrian University of Athens, ‘Ippokrateion’ General Hospital, Athens, Greece; 4 Biochemistry, Biochemical Analysis & Matrix Pathobiology Research Group, Laboratory of Biochemistry, Department of Chemistry, University of Patras, Patras, 26504 Greece; 5 Department of Radiation Oncology, Medical School, University of Patras, Patras, 26504 Greece; 6 Department of Pathology-Cytology, Hospital, Athens, 401 GA Greece

**Keywords:** Head and neck squamous cell carcinoma, Diagnosis, Histopathology, Image-based profiling, Molecular profiling, Metastasis

## Abstract

The incidence of head and neck cancer (HNC), constituting approximately one in ten cancer cases worldwide, affects approximately 644,000 individuals annually. Managing this complex disease involves various treatment modalities such as systemic therapy, radiation, and surgery, particularly for patients with locally advanced disease. HNC treatment necessitates a multidisciplinary approach due to alterations in patients’ genomes affecting their functionality. Predominantly, squamous cell carcinomas (SCCs), the majority of HNCs, arise from the upper aerodigestive tract epithelium. The epidemiology, staging, diagnosis, and management techniques of head and neck squamous cell carcinoma (HNSCC), encompassing clinical, image-based, histopathological and molecular profiling, have been extensively reviewed. Lymph node metastasis (LNM) is a well-known predictive factor for HNSCC that initiates metastasis and significantly impacts HNSCC prognosis. Distant metastasis (DM) in HNSCC has been correlated to aberrant expression of cancer cell-derived cytokines and growth factors triggering abnormal activation of several signaling pathways that boost cancer cell aggressiveness. Recent advances in genetic profiling, understanding tumor microenvironment, oligometastatic disease, and immunotherapy have revolutionized treatment strategies and disease control. Future research may leverage genomics and proteomics to identify biomarkers aiding individualized HNSCC treatment. Understanding the molecular basis, genetic landscape, atypical signaling pathways, and tumor microenvironment have enhanced the comprehension of HNSCC molecular etiology. This critical review sheds light on regional and distant metastases in HNSCC, presenting major clinical and laboratory features, predictive biomarkers, and available therapeutic approaches.

## Introduction

The broad term head and neck cancer (HNC) describes cancers that manifest in the oral cavity, pharynx, larynx, thyroid, paranasal sinuses, nasal cavity and salivary glands. Two-thirds of the estimated 644,000 new cases of HNC each year are diagnosed in developing countries. About 350,000 estimated cancer deaths are HNC-related, while HNC is the sixth most prevalent type of cancer [1]. Head and neck squamous cell carcinoma (HNSCC), accounting for approximately 90% of all HNCs, mainly derived from the mucosal epithelium in the oral cavity, pharynx and larynx [2].

**Fig. 1:**
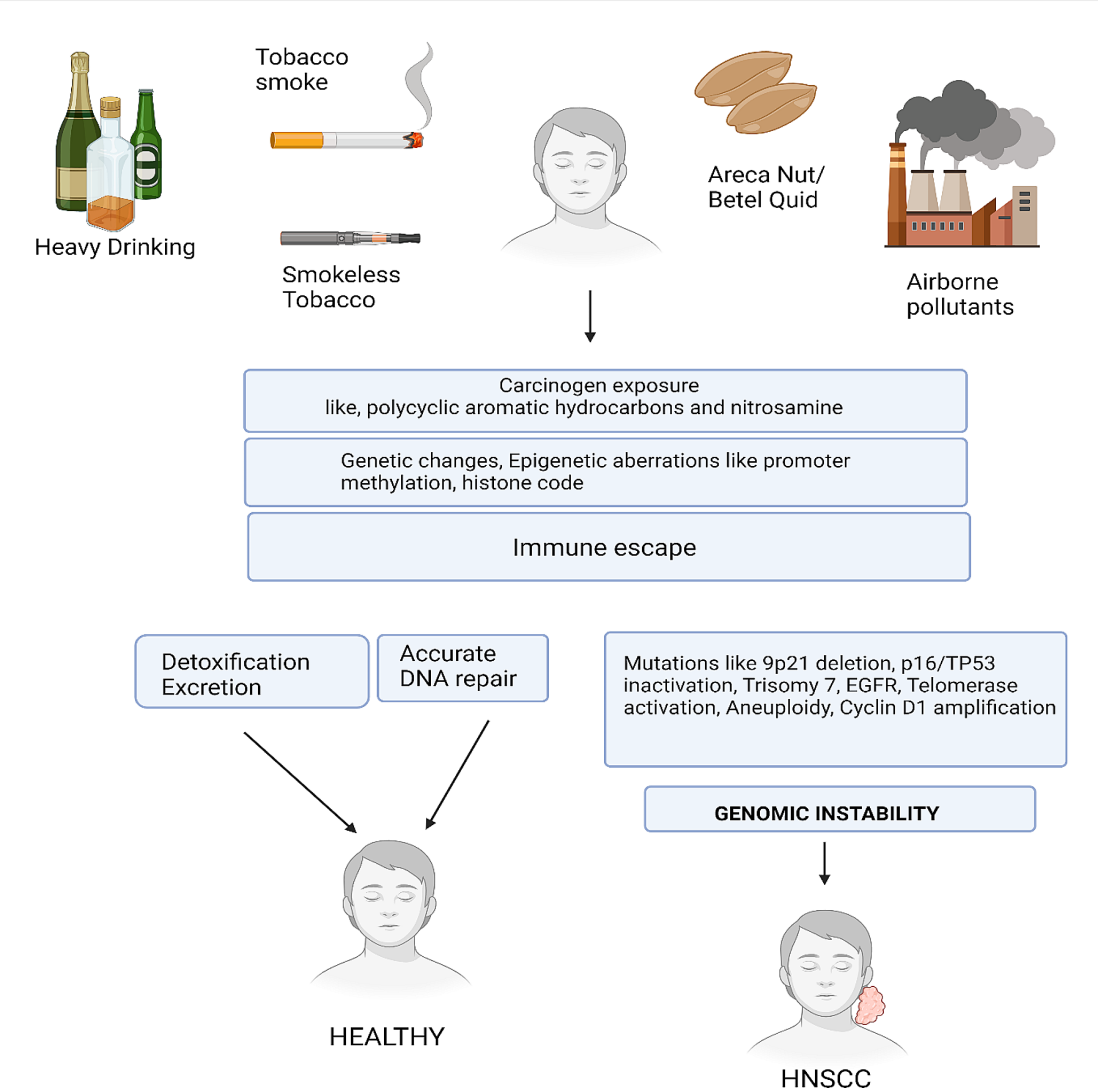
Development of carcinogen-associated HNSCC. A list of etiological risk factors, such as exposure to carcinogens, genetic mutations, and epigenetic aberrations that may cause head and neck squamous cell carcinoma (HNSCC). Detoxification and/or accurate DNA repair sustain homeostasis. However, specific and critical aspects (shown in the right bottom panel) may contribute to genomic instability and development of HNSCC. Created with BioRender.com

Approximately 10% of patients usually appear with a late-stage HNSCC that typically involves local and regional lymph nodes metastasis (LNM) which then could progress to distant metastasis (DM) [3]. The entire aerodigestive tract may be affected by secondary tumors, which can develop at 3–5% annual rates [4]. Patients with locally advanced HNSCC are at least 50% more likely to experience loco-regional relapses or DM, which are typically identified within the first two years of treatment [5]. Either another mucosal site or the location of a previously recognized dysplastic lesion, the reported risk of invasive cancer varies widely between 10 and 40% and is dependent on the histology and duration of follow-up [6]. Depending on the site of the primary tumor, there are different chances of developing a new, secondary tumor, however, the risk is higher for individuals who use tobacco and alcohol [7] (Fig. 1). One of the key elements determining HNSCC prognosis is the condition of the regional neck lymph nodes. Notably, even one positive metastatic lymph node can reduce the chances of survival by as much as 50% [8]. According to studies, 15% of patients with HNSCC who were N0 (negative for neck lymph nodes) during therapy but still were diagnosed HNSCC-positive have been found to have clinically evident DM [9]. Tumor diffusion occurs either by hematogenous spread to distant organs or by lymphatic spread to lymph nodes at local, regional or distant sites. In contrast to other histological types of HNC (e.g. adenoid cystic carcinoma), which can manifest late DM even more than 20 years after diagnosis, HNSCC presents DM shortly after treatment. In a study by Duprez et al. [10], 70% of the patients with DM were diagnosed within 1 year after treatment and 89% within 2 years, the lungs being the most predominant site. The risk of DM is intricately linked to various factors, including mainly the tumor site and size, the nodal status, and the histological grade. Moreover, lymph node extracapsular spread, locoregional residual disease and human papillomavirus (HPV) status, are all associated with an increased likelihood of DM in univariate and multivariate analysis [11]. These factors collectively underscore the complexity of metastatic progression in HNSCC and emphasize the importance of comprehensive risk assessment and management strategies.

LNM and DM of a primary tumor can be detected by several methods. Biomarker detection in liquid biopsy is the most promising non-invasive technique for monitoring metastatic spread during therapeutic response in HNSCC [12, 13]. Moreover, tumor biomarkers can be used in research to evaluate the cellular lineage and histogenic origin of various and distinct neoplasms and produce successful results [14]. However, to comprehend the metastatic process, a number of combined strategies have been used. Combining protein profiling and gene expression studies along with various systems biology approaches enabled the identification of pathways that play a role in the invasive mechanisms of HNSCC [15]. Histopathological image features and machine-learning algorithms can be used to predict somatic mutations, transcription and methylation subtypes, and prognosis of HNSCC [16]. Similarly, computed tomography and magnetic resonance imaging findings have demonstrated unique pathological and clinical characteristics according to HPV-status in HNSCC [17].

In this article we critically present and discuss on the current insights on clinical, imaging and pathological characteristics of HNSCC during LNM and DM. Moreover, we focus on the mechanistic insights on HNSCC progression and the fundamental role of molecular targeting in establishing effective therapeutic approaches.

## Metastatic events and signaling pathways in HNSCC

There are numerous processes involved in metastasis. Major steps include tumor cell invasion into nearby tissue, endothelial transport of cancer cells into vessels, survival in the circulatory system, evasion of the immune system and establishment of a microenvironment conducive to colonization in distant organs [18]. The coordination of all these events is crucial to prevent the complete failure of this complex process. The preference of cancer cells for colonization in particular organs is a critical consideration [19]. In models of spontaneous metastasis, cancer cells proliferate in the circulatory system to maintain viability, are released, and form colonies near secondary sites. However, they often fail to replicate normal metastasis and instead develop niche pre-metastasis from specific growth of the primary tumor [20, 21]. Tumor budding is recognized as the initial histopathological event in metastasis and plays a pivotal role in its onset [22]. The significant correlation between high-grade budding and metastasis in various carcinomas, including different subsites of HNSCC, suggests the migratory ability of tumor buds [23, 24]. Aldehyde dehydrogenase 1 (ALDH1), a characteristic biomarker of cancer stem cells, is highly expressed in tumor budding cells in nasopharyngeal carcinoma. This observation raises the possibility that these budding cells possess the invasive and metastatic properties of cancer stem cells [25].

**Fig. 2:**
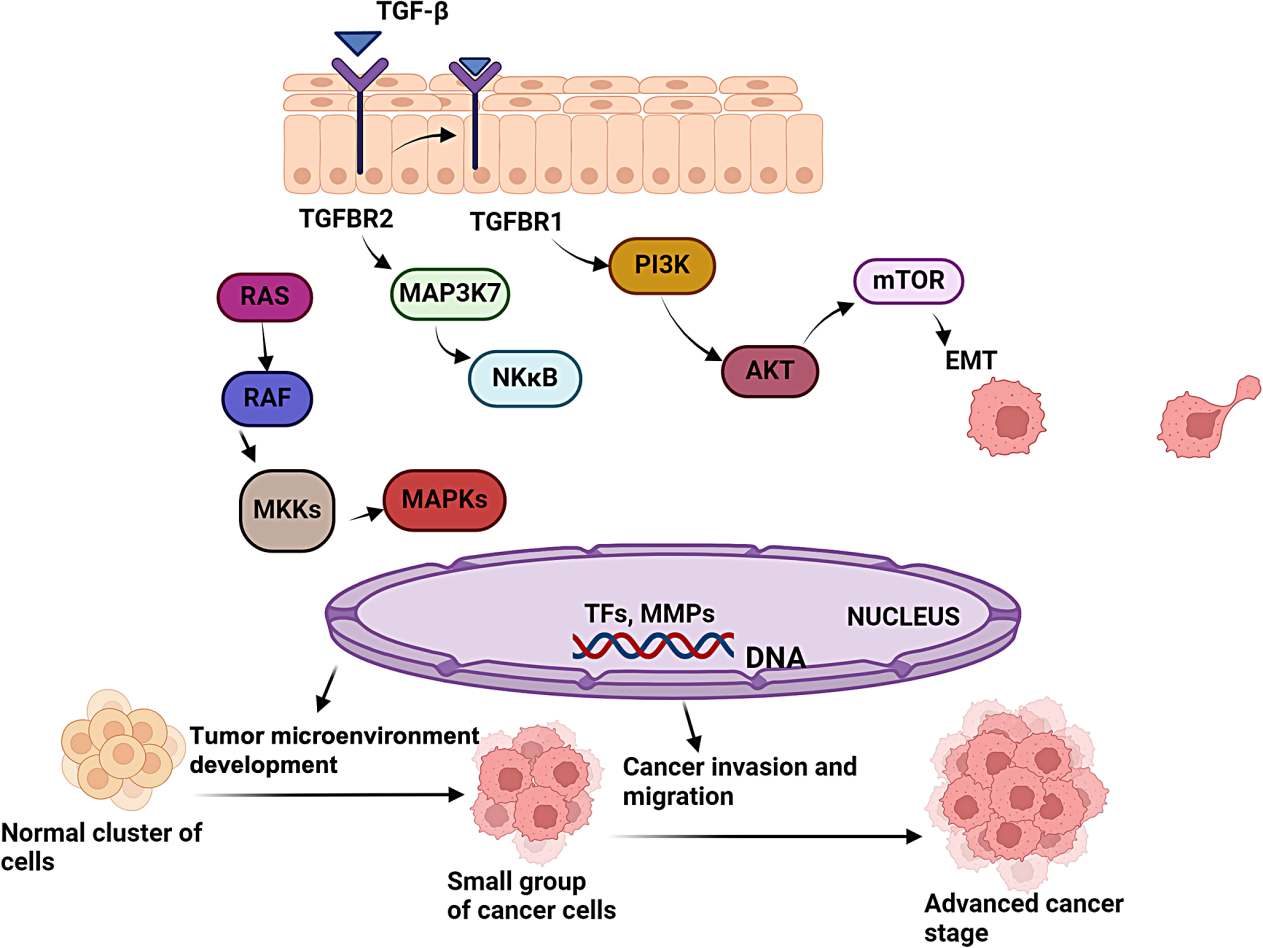
Pathways in HNSCC cells involving growth factor receptors like TGFβ causing cell migration and invasion leading to metastasis. MKKs consist enzymes that phosphorylate and activate MAPKs, serving as intermediaries in cellular signalling pathways. MAPKs are protein kinases that regulate gene expression and cellular processes in response to extracellular stimuli through phosphorylation of target proteins. MAP3K7 is a protein kinase that activates downstream MKKs in the MAPK signalling pathway, involved in diverse cellular processes such as immune responses and inflammation. Abbreviations: TGFβ, transforming growth factor beta; TGFBR, transforming growth factor beta receptor; RAS, rat sarcoma virus; RAF, rapidly accelerated fibrosarcoma; MEK, mitogen-activated protein kinase kinase; MKK, MAP kinase kinase; MAP, mitogen-activated protein kinase; MAP3K7, mitogen-activated protein kinase kinase kinase 7; NF-κΒ, nuclear factor kappa B; PI3K, phosphoinositide 3 kinase; AKT, protein kinase B; mTOR, mammalian target of rapamycin; EMT, epithelial-to-mesenchymal transition; TFs, transcription factors; MMP, matrix metalloproteinase; DNA, deoxyribonucleic acid. Created with BioRender.com.

**Fig. 3:**
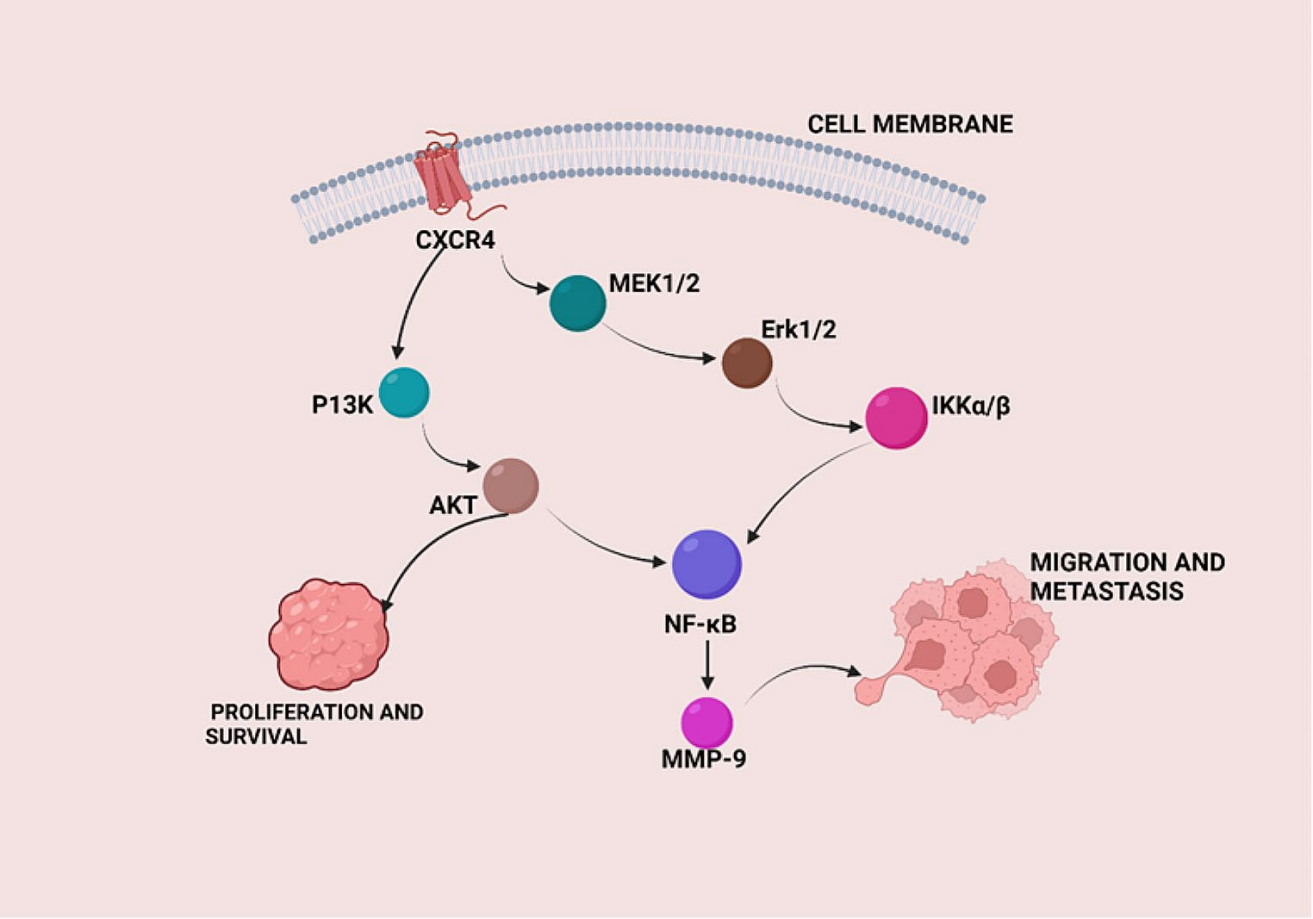
Pathways in HNSCC cells involving growth factor receptors like CXCR4 causing cell migration and invasion leading to metastasis. Abbreviations: CXCR4, C-X-C chemokine receptor type 4; PI3K, phosphoinositide 3 kinase; AKT, protein kinase B; MEK, mitogen-activated protein kinase kinase; ERK, extracellular signal-regulated kinase; IKKα/β, IκB Kinase α/β; NF-κB, nuclear factor kappa B; MMP-9, matrix metalloproteinase 9. Created with BioRender.com.

Several interlinked signaling cascades are often mediating cell behavior and prognosis in HNSCC [26]. These mainly involve cytokine signaling including the activation of TGF-βR (Fig. 2) and CXCR4 (Fig. 3), which mediate the functions of several extracellular matrix (ECM) regulators that enforce intracellular signaling enhancing cell migration and invasion leading to metastasis [27]. The TGF-βR pathway plays a vital role in the spread of HNSCC. In HNSCC, aberrant TGF-β signaling is common and fuels the tumor’s growth [28]. TGF-β can trigger a process called epithelial-mesenchymal transition (EMT), boosting the aggressive phenotype of cancer cells that move and invade more easily, thus facilitating the initiation of metastasis [29]. Although the concept of EMT is widely debated and its validity is not yet confirmed [30], it is still regarded as an important and advanced theory for the study of cancer metastases. Moreover, TGF-β signaling controls the activity of genes like E-cadherin and vimentin that are involved in cell adhesion, movement and invasion, and generates a group of cancer cells that are highly mobile and can spread rapidly to other parts of the body [31, 32].

CXCR4 plays an important role in the spread of HNSCC. CXCR4 is a receptor that cooperates with CXCL12 to boost cancer cell motility, invasion and metastasis. Recent studies shed light on how this pathway works in HNSCC spreading. There was a significant increase in CXCR4 mRNA levels in highly metastatic cells compared to cells with less aggressive characteristics. In HNSCC, CXCR4 was linked to EMT markers, indicating that CXCR4 levels correlate closely with aggressive tumors, poor prognoses and disease spread. Additionally, the communication between HNSCC cells and lymphatic endothelial cells (LECs) was seen to promote tumor spread through CXCL5-CXCR2 signaling [33–35].

**Fig. 4:**
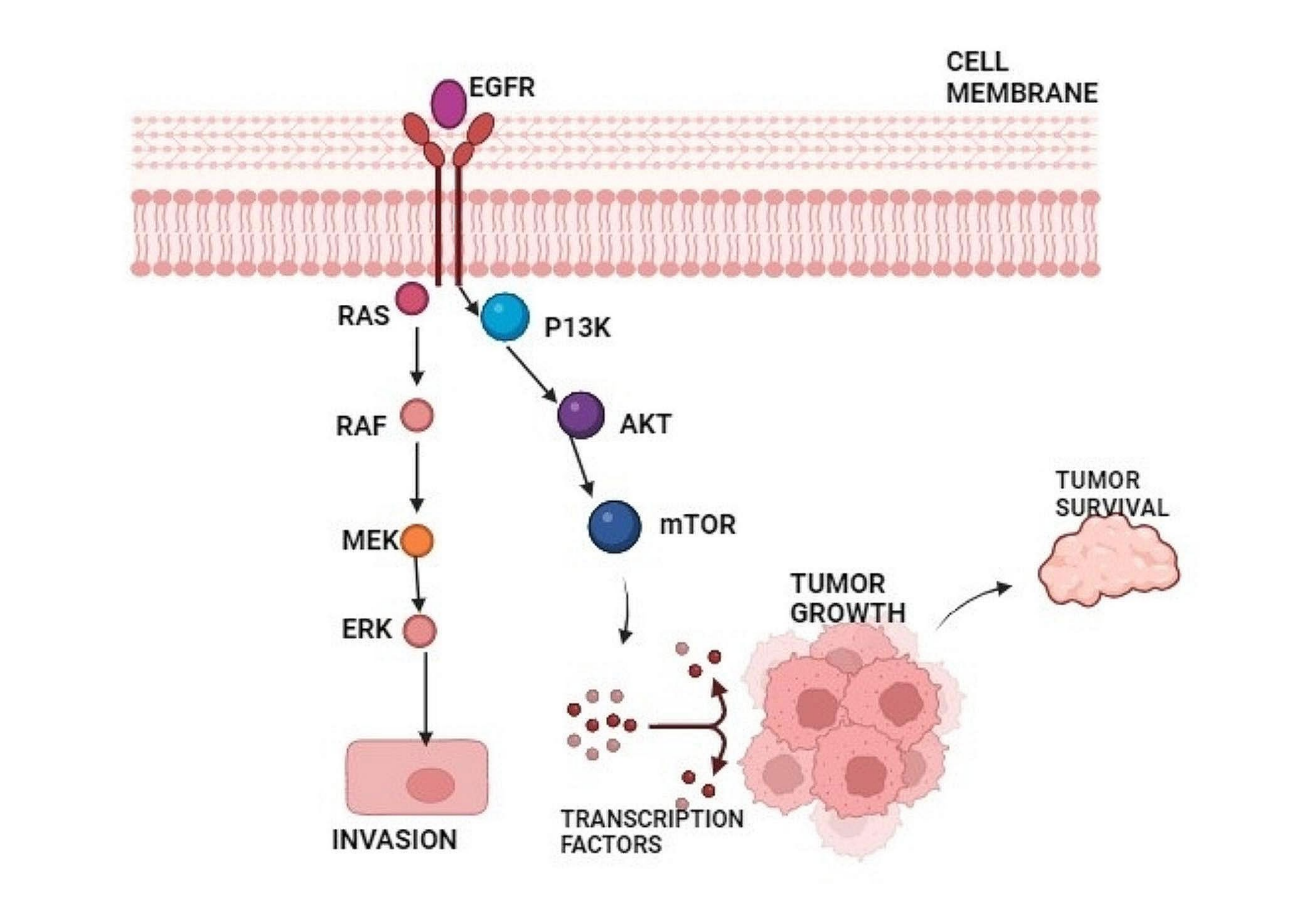
EGFR-evoked signaling pathways related to HNSCC invasion to adjacent tissues. Abbreviations: EGFR, epidermal growth factor receptor; RAS, rat sarcoma virus; RAF, rapidly accelerated fibrosarcoma; MEK, mitogen-activated protein kinase kinase; ERK, extracellular signal-regulated kinase; PI3K, phosphoinositide 3 kinase; AKT, protein kinase B; mTOR, mammalian target of rapamycin. Created with BioRender.com.

In HNSCC, multiple pathways activate the epidermal growth factor receptor (EGFR) (Fig. 4). When EGFR interacts with epidermal growth factor (EGF), heparin-binding EGF (HB-EGF), amphiregulin, transforming growth factor alpha (TGFα), epiregulin, and betacellulin, which are ligands of the HER family, a signal transduction cascade is initiated, activating numerous pathways simultaneously [36]. EGFR’s significance in various human tumors, particularly HNSCC, is crucial [37]. In these tumors, EGFR often becomes hyperactive, leading to downstream chaos, particularly in the PI3K/Akt/mTOR pathway. This disruption fuels the progression of HNSCC, promoting angiogenesis, tumor growth, invasion and metastasis [38–40]. EGFR serves as the primary driver of invasion in HNSCC. Activation of EGFR by triggers like EGF initiates a cascade of events that drive further invasion. This activation triggers pathways such as MAPK and PI3K, essential for cell growth, survival and motility. In HNSCC, EGFR promotes invasion through various mechanisms: enhancing cellular mobility by altering cell morphology, increasing MMP-9 to break down barriers, inducing an invasion-ready state (EGFR-EMT), and cooperating with pathways like c-MET/HGF for cell growth, invasion, and angiogenesis [41–43].

Certain signaling pathways, such as PD-1/PD-L1 and STAT3, have an impact in HNSCC associated with immune evasion, which is a fundamental mechanism in cancer evolution. Programmed death receptor-1 (PD-1) and its ligand PD-L1, play a vital role in immune evasion by HNSCC. A PD-1 - PD-L1 interaction results in T cells exhaustion and inhibition of the anti-tumor immune response. This pathway is often upregulated in HNSCC, permitting tumor cells to evade immune surveillance [44]. Signal Transducer and Activator of Transcription 3 (STAT3) is a transcription factor that is commonly activated in HNSCC. By upregulating the expression of immunosuppressive molecules such as PD-L1, TGF-β1, VEGF, IL-6 and IL-10, STAT3 promotes tumor growth and immune evasion [45, 46]. Moreover, STAT3 activation in tumor-associated immune cells leads to anti-tumor immune suppression [47].

Finally, the PI3K/AKT/mTOR pathway is also significant for immune evasion in HNSCC by promoting tumor cell survival, inducing immunosuppressive factors, inhibiting antigen presentation and amplifying regulatory T cell function [48, 49].

## Clinical factors associated with the metastatic potential of HNSCC and the role of imaging techniques

During diagnosis, 2/3 of HNSCC nurture micro-metastasis and present local invasion. Despite advancements in cancer detection and treatment, residual signs of disseminated disease may persist, leading to tumor invasion and loco-regional or distant metastases, even after effective treatment of the primary tumor. Micro-metastases, which are multicellular secondary cancer cell clusters, often evade detection in clinical diagnostic scans and can remain active, representing a potential manifestation of this residual disease component [50].

Lymph node metastasis (LNM) is a well-known and clinically recognized prognostic factor for HNSCC and other solid malignancies [51]. In HNSCC regional LNM indicates aggressive tumor biology and acts as a location of subsequent DM [8, 52]. The metastatic spread of HNSCC to neck lymph nodes has been mainly documented at levels III and IV, sometimes with no discernible engagement at levels I and II. In addition, 5% of patients demonstrated “peppering” at multiple lymph node levels without any obvious macroscopic involvement [53]. About 10% of individuals with HNSCC may develop DM with lungs, bones and mediastinal lymph nodes being the most frequent and typical sites [54–56]. DM in HNSCC represent a complex and critical aspect of disease progression, which significantly impact survival and treatment options.

SCC was the most frequent histological type discovered in metastatic neck lymph nodes, according to all research conducted on the prevalence of metastatic disease in HNC patients. From HNC primary sites, SCC quickly proliferate [57]. The normal anatomical lymphatic pathways either delay or completely ignore the order in which nodal metastasis should develop. Clinical observations revealed that the involvement of the metastasis of various regions typically advances from the higher to the lower region of the neck in HNSCC, and this pattern can also be recognised in normal lymphography. In patients enduring HNSCC and non-HNSCC, the hazy movement of tumour cells escaping from typical lymph nodes that drain to other lymph nodes has been recorded or well documented [58].

The tumor-node-metastasis (TNM) staging, which is currently the most accurate indication of a patient’s prognosis and the elements that define the stage of disease progression as well as the course of treatment, takes into account both the main tumour dimension and LNM status [59]. Due to the low prevalence of DM at presentation, it is crucial to establish criteria for choosing people whose DM status should be assessed [60]. Greater nodal stage, involvement of the lower neck lymph nodes, and frequency of lymph node metastases are all clearly associated with the development of DM [61–64]. People at risk for DM and those in whom it is important to rule out such metastases typically undergo laboratory tests, computed tomography (CT) of the lungs, bone scintigraphy, and ultrasound (US) or CT of the liver due to the fact that the lungs, bone, and liver are the most prevalent locations of DM [65].

Patients with HNSCC represent almost two thirds of those with advanced cancer, as opposed to only a third of those with early-stage disease [66]. The likelihood of neighboring lymph nodes being infected by locally invasive HNSCCs is higher than the likelihood of hematogenous dissemination [67]. Additionally, the microenvironment of the primary site may have a different tumour ecology and phenotype than the metastatic LNs [68, 69]. More significantly, it has been revealed that tumor cells respond to treatment differently in various microenvironments [70, 71]. Therefore, growing evidence suggests that in order to determine the best course of treatment and enhance prognosis, the evaluation of cancer patients should be enhanced based on the underlying tumour and metastatic microenvironment. Surface measures alone have historically been used to stage primary tumors of the oral cavity. Depth of invasion (DOI) is now included in the T staging of primary tumours because of its prognostic impact on the likelihood of concealed metastases and disease-specific survival [72, 73]. Each successive edition of the TNM system has evolved as non-anatomic prognostic features have been introduced, and there has also been a concurrent recording of more prognostic variables and new information that isn’t yet appropriate for the staging criterion. Table 1 shows the tumor classification (T) for oral cancers according to the extent and DOI of primary tumor [74].

**Table 1:** T classification of oral cancer, 8th edition [74]

Tumor category	Tumor characterization
TX	Primary tumor cannot be examined
Tis	Carcinoma in situ
T1	Tumor ≤ 2 cm, ≤ 5 mm DOI
T2	Tumor ≤ 2 cm, DOI ≤ 5 mm and ≤ 10 mm or tumor > 2 cm but ≤ 4 cm and ≤ 10 mm DOI
T3	Tumor > 4 cm or any tumor > 10 mm DOI
T4	Moderately advanced or very advanced local disease
T4a	Tumor involves adjacent structures, such as the cortical bone, deep extrinsic muscles of the tongue, maxillary sinus, or skin of the face.
T4b	Tumor involves masticator space, pterygoid plates, or skull base and/or encases the internal carotid artery.

Imaging techniques can provide accurate non-invasive soft tissue characterization for assessing superficial primary sites and nodal basins. In head and neck imaging, it is possible to detect neck tumours, lesions of the salivary glands, thyroid nodules, and variations in the morphology of adjacent lymph nodes [75]. The primary site of HNC, nodal disease, and staging using the well-established TNM staging approach are commonly assessed with CT. The infra-hyoid neck may be evaluated well with minimal artifact movement with CT because of its rapid acquisition time. It may simultaneously check the thorax for lung metastases, synchronous primary lung lesions, paratracheal and upper mediastinal lymph nodes and more.

Since there are many neck lymph node levels to analyze and a variety of methodologies and criteria are given for metastatic lymph nodes, the assessment of imaging of lymph node metastasis in HNSCC is a significant difficulty for a radiologist conducting research on this problem. After analyzing all the predictors possible for metastasis of the lymph nodes on CT images, it was seen that the diameter of the lymph node was categorized in 2 ways: axial and coronal diameters as the shortest and longest, respectively [76]. The additional potential factors were long to short axis ratio, necrosis presence, lymph node conglomeration, primary tumor T-stage, etc. The definition given for necrosis was described as low density at the central position with irregular or circular rim kind of lymphatic tissue that was left as a residue [77, 78]. Descriptions of neighboring soft tissue infiltration included a poorly defined nodal region or infiltration into the muscles or fat strands in the neck. The overall extent of necrosis was categorized into several types: absence, presence, and cystic presence. By doing a visual analysis of all 3 types of degree of necrosis, it could be seen that a rim-like structure emerged with > 90% low density [79–81].

A study by Morisala et al., aimed to identify CT imaging characteristics of sub-centimeter lymph nodes in oral SCC patients with clinically negative necks (cN0) to predict the likelihood of nodal metastases on histopathology. Retrospective review of patients undergoing elective neck dissection (END) demonstrated that round/oval shape, asymmetric number and disrupted fatty hilum of lymph nodes on pre-operative CT imaging were highly predictive of occult nodal disease. These findings provide valuable insights for guiding decision-making regarding END versus clinical surveillance in cN0 OCSCC patients [82]. In another study, Fujita et al. [17] aimed to explore imaging characteristics of nodal metastasis by HPV status in HNSCC and their influence on outcomes. CT and MRI data from 139 HNSCC patients were retrospectively reviewed. They found that in HPV-positive HNSCC, nodal metastases were notably more prevalent, whereas HPV-negative HNSCC exhibited a higher incidence of disease recurrence. Despite the majority of HPV-positive patients with nodal metastasis presenting extracapsular spread (ECS), their recurrence rates were comparatively lower than those of HPV-negative patients. These findings suggest a distinct pathological pattern and clinical behavior between HPV-positive and HPV-negative HNSCC.

Advanced functional MRI techniques, such as diffusion and perfusion imaging techniques, can also be used to detect metastatic LNs [83, 84]. Increased cell density during LNM may modify water diffusivity, which may be assessed by apparent diffusion coefficient (ADC) utilizing diffusion-weighted MRI (DWI-MRI) [83]. To evaluate tumor vascularity, dynamic contrast-enhanced (DCE)-MRI has been routinely employed. In contrast, positron emission tomography (PET) imaging with 18 F-fluorodeoxyglucose (18 F-FDG) shows inconsistent specificity (77–93%) but excellent sensitivity (92–100%) in identifying nodal metastases [85, 86]. Previous research looking at combining PET and MR imaging discovered that doing so might increase the sensitivity and specificity of finding LNM in HNSCC [87].

## Histopathological features and metastatic behavior

Histopathological evaluation of HNSCC provides a microscopic glimpse into key tumor traits. These characteristics provide vital information about the tumor’s behavior, prognosis and potential treatment options. Among the notable histopathologic features is the tumor grade, which indicates how closely the cells resemble normal ones and plays a significant role in prognosis. According to tumor differentiation, patients with well-differentiated (WHO grade 1) and moderately-differentiated (WHO grade 2) tumors had better survival compared to poorly-differentiated (WHO grade 3) tumors [88]. The tumor’s size is another key determinant of cancer stage and propensity to spread. The invasive nature of HNSCC, infiltrating neighboring tissues like muscle, bone and blood vessels, critically influences prognostic outcomes. Furthermore, the infiltration of lymphatic and blood vessels increases the risk of metastasis to both nearby lymph nodes and distant locations. The invasion of nerves by HNSCC tumors not only causes pain, but also affects available treatment options. Surgical margins that reveal residual tumor cells after surgery indicate incomplete tumor removal, which increases the risk of recurrence. Moreover, the presence of immune cells within the tumor’s microenvironment may provide insight into the immune response against the tumor, potentially holding prognostic value [89–91].

The term “lymphatic invasion” refers to an invasion of a vessel’s tunica media together with intimal ulceration, while the existence of tumor cell aggregation inside endothelium-lined regions devoid of underlying muscle walls was referred to as vascular invasion [91]. A study by Adel M et al., was conducted in 571 patients (55 women and 516 men) with oral SCC (OSCC) aged at diagnosis from 21.9 to 86.8 years (median, 51.2 years). There was no evidence that lymphatic invasion was related to a specific sex or age group. Considering vascular invasion, a similar conclusion was reached for sex and age. Pathological differentiation, nodal metastases (*p* = 0.009), extracapsular dissemination, perineural invasion, and bone invasion all showed statistically significant relationship with lymphatic invasion, and tumor classification (T). Significant relationships between vascular invasion and T classification, extracapsular spread, nodal metastasis, perineural invasion, depth of invasion, and pathologic differentiation were also found. However, there was no significant relationship between vascular invasion and bone invasion. Overall, these correlations showed that the presence of neck metastasis, perineural invasion, extracapsular dissemination, poor differentiation, and deeper tumor depth was positively correlated with the histological findings of lymphatic and vascular invasion in the main OSCC tumors [92].

In order to properly detect and diagnose a tumour, one must have a comprehensive understanding of the tumour’s cellular, biochemical, molecular and pathophysiological aspects. Even though the undifferentiated tumour is still an issue, these patterns help the histopathologist make an accurate diagnosis. It is important to note that these histological patterns are not necessarily diagnostic, as variants and subpatterns can also be observed. Based on patterns found on hematoxylin and eosin-stained sections, the diagnosis may then be confirmed using certain stains, immunohistochemistry, and various molecular diagnostic methods [93]. It was also noted that the majority of OSCC patients with lymphatic or vascular invasion required postoperative adjuvant radiation or chemoradiotherapy since these illnesses were found to be closely related with a number of clinical variables [92].

Clinical and histological assessments remain the main prognostic methods in standard medical practice. Histological characteristics such as perineural invasion, vascular invasion, and the degree of differentiation are widely used as prognostic indicators when assessing patients with HNSCC [94–96]. The composition of tumour cells, the presence of stromal components, local immunological responses, and necrosis, are all factors that histological investigation can provide significantly more in-depth information about, all of which may be important prognostic indicators. Due to their predictive value in many types of cancer, these histological features are currently used in clinical practice [97–99]. When examining relationships with clinicopathological parameters in HNSCC, tumour necrosis and tumour budding were also found to be substantially correlated with tumour size and clinical stage [100]. In order to obtain additional histological information on the aggressiveness of HNSCC, assessment of tumour size to determine tumor/stroma ratio is a straightforward, reasonably priced procedure that may be utilised in any standard pathology clinic.

## Molecular profile of the primary tumor and metastatic spread

HNSCC lesions which are a prevalent model for cancerization, allow for the development of numerous prognostic markers [101, 102]. Molecular pathologic features of HNSCC may differ greatly depending on the location and etiology of the disease [103]. The molecular and genetic characteristics of cancer cells may be the main predictors of metastasis in HNSCC, accompanied by indications of aggressive lymphatic and blood vessels spreading inside the primary tumor [104]. The links between genome alterations and patterns of dissemination in metastatic disease were found to be present in more than 50 types of tumors. This was done by analyzing genomic and clinical data from cohorts of more than 25,000 patients suffering from DM [105]. The most prevalent kind of genetic alteration in HNSCC is tumor-suppressor TP53 mutation which is present in almost 70% of cases [106]. *TP53* mutations are often associated with immunotherapy and chemotherapy and could be used as a specific predictor of treatment response in HNSCC patients [107]. HNSCC has also been associated with mutations in other known oncogenes and tumor suppressors such as Cyclin D1, NOTCH1, PIK3CA, MYC, CDKN2A, PTEN and F-box/WD repeat-containing protein 7 [108, 109]. Moreover, various HNSCCs have been linked to a high prevalence of HRAS mutations, copy number changes, and abnormal expression levels of KRAS, NRAS, MYC and EGFR [108]. The genomic analysis of 110 Indian patients with HNSCC identified 5 additional commonly mutated (10–22% of patients) genes linked to OSCC-GB: USP9X (Ubiquitin-Specific Peptidase 9X), MLL4 (Mixed-lineage leukemia 4), ARID2 (AT- rich interactive domain-containing protein 2), UNC13C (UNC-13 homolog C), and TRPM3 (Transient receptor potential cation channel subfamily M member 3) [110]. Furthermore, three others genes WNT16, PARP1 (Poly (ADP-ribose) polymerases), and ATF4 (Activating Transcription factor 4) have a mutation frequency of about 50%. Since oral cancer is recognized for its high expression levels of PARP1, the mutation frequency exceeding 30% suggests that activating mutations are present in all tumor locations [111].

To understand molecular profiling techniques and approaches in HNSCC, we need to be aware of some approaches such as NGS (Next-Generation Sequencing) technology that includes targeted gene panels, whole genome sequencing and whole exome sequencing. NGS is limited to some specific genes, but it can also concentrate on regions that code for base pairs of the entire genome or it can also include the complete analysis of the tumor genome, which includes intronic regions [112]. Many studies have shown mutations that happen at the somatic hotspots get involved in tumorigenesis and are titled as drivers, which may be recurrent mutations occurring in areas of the genome. In some cases where the aberration in the target molecule occurs occasionally, it can be a challenging task taking patients suffering from rare mutations to clinical trials and can act as a barrier to the development of upcoming drugs on the market. A tumor labeled by intratumoral heterogeneity may have an impact on the efficiency of a therapy where a specific variation in the molecule can be called a true driver in a specific tumor [113, 114].

The application of NGS techniques and its related methodologies is useful for understanding the integration of clinical, histopathological and molecular data for assessing metastatic risk in HNSCC. The results of an NGS study can also provide a physician with a list of drugs that can be adapted for patients suffering from cancer [115]. In large-scale tumor profiling studies using NGS methods, significant genomic similarities were found between different types of tumors, which actively shared alterations in driver genes. A BRAF mutation, which has been found in many types of tumors, is an example of this scenario [116]. The alterations in the genome don’t always show the way to the activation or addiction of the oncogenic pathway. There are also some cells that become dependent on one cell for activating a specific oncogenic pathway. In spite of high success rates for analyzing molecular structures to detect HNSCC in patients, NGS technology has shown to be more accurate and faster than other approaches. Utilizing artificial intelligence (AI), particularly in the realm of automated image analysis, has emerged as a valuable tool in the diagnostic assessment of HNC, including HNSCC. The application of AI extends to the classification of lymph nodes in locally-advanced HNSCC, presenting promising diagnostic support. A growing body of research explores the potential of AI and machine learning (ML) in enhancing HNC detection across various imaging modalities. These approaches demonstrate the capacity to achieve levels of accuracy surpassing human judgement in data predictions. However, for widespread clinical integration, the necessity for large-scale, multi-centric prospective studies becomes apparent, facilitating the seamless transition of AI technologies into routine clinical practice [117].

A significant prognostic and predictive marker for HNSCC is the presence of HPV. HPV-positive HNSCC constitutes a significant subset, comprising up to a quarter of all HNSCC cases, with primary tumors predominantly originating from the oropharynx, notably the tonsil and base of tongue [118]. Metastasis to cervical lymph nodes often marks the initial presentation of HPV- positive HNSCC and can be detected via fine-needle aspiration, frequently exhibiting cystic characteristics with a non-keratinizing, basaloid morphology. The determination of HPV status in metastatic HNSCC holds substantial therapeutic and prognostic implications, given the more favorable prognosis associated with HPV-positive tumors compared to conventional HNSCC. Therefore, HPV testing is recommended for any SCC of unknown primary identified in neck lymph nodes. Numerous techniques have been used in supplementary research to distinguish between HPV-positive and -negative HNSCC. These techniques include immunohistochemistry (IHC) staining for p16 as a surrogate marker, HPV polymerase chain reaction (PCR) testing for viral DNA or RNA detection, HPV in situ hybridization (ISH) analysis and more recent methods that are actively being researched [119, 120]. As it identifies transcriptionally active HPV, RT-PCR amplification of viral E6/E7 mRNA is currently regarded as the “gold standard” for the diagnosis of clinically relevant HPV infection inside tumour tissues. The technique is trustworthy when used with formalin-fixed paraffin-embedded (FFPE) samples as well as fresh frozen specimens [120]. Besides, for cancers that are HPV-positive, inactivating the p16 gene is a very important target. Examining and learning more about DNA damage repair genes and how destructive they may be is becoming a certain and promising strategy to combat HPV-negative cancers as well. Exploring and understanding etiological features is also another approach to defining the characteristics of the tumor and metastasis [121].

## Emerging therapeutic approaches for HNSCC

Patients with recurrent HNSCC may be candidates for salvage surgery, further radiation, or chemotherapy, however this is not always applicable to patients with DM. Nowadays, various metastatic cancers, including HNSCC, are presently addressed using immunotherapy as a frontline approach, often combined with conventional chemotherapy, targeted therapy or other modalities. Cetuximab, was the first a monoclonal antibody that emerged as a promising immunotherapy option in the management of locally advanced HNSCC. Cetuximab acts by targeting the extracellular domain of EGFR, which along with EGF are upregulated in 90% of HNSCC patients and linked with poor outcomes [122]. Clinical trials have demonstrated its efficacy when combined with standard treatments such radiotherapy or platinum-based chemotherapy. In a study by Bonner et al., the combination of cetuximab and radiation resulted in a 45.6% 5-year overall survival rate compared to a radiation-only (36.4%) approach [123]. Similarly Vermoken et al. reported a prolonged median overall survival from 7.4 months in the chemotherapy-alone group to 10.1 months when cetixumab added to the regimen [124].

In 2016, the anti-PD-1 immune checkpoint inhibitors (ICIs) nivolumab and pembrolizumab were both approved for HNSCC patients with recurrent or metastatic HNSCC. In 2019, pembrolizumab was approved for first-line treatment either as monotherapy in PD-L1 expressing tumors or combined with chemotherapy [125]. ICIs function by prompting the host’s immune system to identify abnormal cells, employing immune cells’ cytotoxic capabilities to target cancerous cells, particularly those specific to tumors and responsive to T cell cytotoxicity. T cells activate tumor cells via a two-way signaling pathway: the first signal involves T cell receptor recognition of tumor cells presenting major histocompatibility complex (MHC) antigens, while the second signal results from interaction with co-stimulatory factors and B7 molecules on antigen-presenting cells (APCs) [126]. Without both signals, T cell activation is hindered. Immune checkpoints regulate immune response initiation and cessation, as well as T cell activation and other host immune activities [127]. Immunotherapeutic approaches may necessitate a distinct evaluation compared to standard anti-tumor treatments. Patients receiving ICIs may experience hyper-progression characterized by rapid tumor growth or pseudo-progression, an initial tumor enlargement followed by regression [128]. Hyper-progression correlates with poor survival and may result from an excessive immune response, PD-1 mediated tumor promotion or familial cancer predisposition. Pseudo-progression may elucidate enhanced chemotherapy efficacy post anti-PD-1 therapy failure. Comparing nivolumab to a three-component cetuximab, docetaxel, and methotrexate regimen during disease progression, nivolumab exhibited a complete survival scale of 7.7 months [129]. Reduction relative to nivolumab could stem from crossover effects in the chemotherapy group, where patients often receive immunotherapy with secondary effects. Additionally, the less favorable outcomes with weekly nivolumab compared to three-weekly docetaxel, commonly used due to its efficacy, may contribute to the discrepancy [129]. Today, both nivolumab and pembrolizumab, were found to be effective in platinum sensitive patients with recurrent or metastatic HNSCC [130].

Although chemotherapy is considered the standard treatment for metastatic HNSCC, radiation techniques, such as stereotactic body radiotherapy, can also provide palliation. After careful patient selection, high-dose regimens could be used in metastatic patients with good performance status. In addition, oligometastatic disease can be treated more effectively in some clinical settings [131]. However, there are relatively few clinical and radiological indicators of radiation therapy toxicity and response to treatment, and they are not frequently employed in clinical practice for head and neck tumors. One of the most exciting areas of study is the “omics” method. It might generate a lot of data that could be used to forecast patient toxicity and the response to radiation treatment [132].

Clinical experiments conducted over the past ten years have demonstrated the prognostic role of numerous molecules involved in tumor metastasis and growth. None of them have received much attention in clinical practice up to this point, most likely because research on their expression, typically using quantitative or immunohistochemical methods, has shown that these molecules are involved in the development of tumors without being able to conclusively attribute to them a specific marker function for HNSCC metastases. Clinicians may have the chance to develop novel diagnostic strategies, therapeutic integration, and meticulous follow-up if a significant molecular marker that can predict cervical lymph node metastasis from a primary tumor biopsy is found. This is in addition to the conventional clinicopathological parameters. The follow up care and strategies for HNSCC can be classified into 2 types which are standardized and personalized, respectively. The predetermined therapy program for HNSCC patients over a period of approximately five years essentially constitutes the typical follow-up [133]. The Bowen’s key method is used to determine whether it is feasible to offer a standardized or personalized follow up to a HNSCC patient. It suggests that a specific framework of feasibility studies is present in the areas of focus to treat such a patient [134]. Data collection, management, and a certain sample size are additional requirements for validating the personalized treatment plans and follow-up strategies for HNSCC. One of the surveys showed that 25% of HNC patients chose personalized follow up after making a decision [135].

## Conclusions and future perspectives

The ability to detect the presence of micro-metastases or the metastatic potential of a tumour at an early stage might influence the development of metastatic tumours and the treatment strategy. Numerous potential applications in HNSCC treatment have been revealed through invasion studies. The simplest strategy is to develop inhibitors directed at molecules crucial for invasion. Today, clinical and histological assessment of the tumor is still used to predict HNSCC metastatic risk. It has also been made possible by developments made in the past ten years in understanding the molecular pathways involved in neoplastic tumour formation to identify molecules that could be used as potential prognostic indicators for this malignancy. Sometimes the patterns of certain genomic alterations vary among patients. Clinical parameters must also be tested to ensure the quality of drug screening assays. Comprehending forthcoming advancements in HNSCC technology, along with enhancing capabilities to detect biomarkers and evaluate medications, could significantly enhance the management of this malignancy.

## Data Availability

No datasets were generated or analysed during the current study.
